# Neurodevelopmental effects of chronic exposure to elevated levels of pro-inflammatory cytokines in a developing visual system

**DOI:** 10.1186/1749-8104-5-2

**Published:** 2010-01-12

**Authors:** Ryan H Lee, Elizabeth A Mills, Neil Schwartz, Mark R Bell, Katherine E Deeg, Edward S Ruthazer, Nicholas Marsh-Armstrong, Carlos D Aizenman

**Affiliations:** 1 Department of Neuroscience, Brown University, Providence, RI 02912, USA; 2 Solomon H Snyder Department of Neuroscience, Johns Hopkins University School of Medicine, Baltimore, MD, USA; 3 Montreal Neurological Institute, McGill University, Montreál, QC, Canada

## Abstract

**Background:**

Imbalances in the regulation of pro-inflammatory cytokines have been increasingly correlated with a number of severe and prevalent neurodevelopmental disorders, including autism spectrum disorder, schizophrenia and Down syndrome. Although several studies have shown that cytokines have potent effects on neural function, their role in neural development is still poorly understood. In this study, we investigated the link between abnormal cytokine levels and neural development using the *Xenopus laevis *tadpole visual system, a model frequently used to examine the anatomical and functional development of neural circuits.

**Results:**

Using a test for a visually guided behavior that requires normal visual system development, we examined the long-term effects of prolonged developmental exposure to three pro-inflammatory cytokines with known neural functions: interleukin (IL)-1β, IL-6 and tumor necrosis factor (TNF)-α. We found that all cytokines affected the development of normal visually guided behavior. Neuroanatomical imaging of the visual projection showed that none of the cytokines caused any gross abnormalities in the anatomical organization of this projection, suggesting that they may be acting at the level of neuronal microcircuits. We further tested the effects of TNF-α on the electrophysiological properties of the retinotectal circuit and found that long-term developmental exposure to TNF-α resulted in enhanced spontaneous excitatory synaptic transmission in tectal neurons, increased AMPA/NMDA ratios of retinotectal synapses, and a decrease in the number of immature synapses containing only NMDA receptors, consistent with premature maturation and stabilization of these synapses. Local interconnectivity within the tectum also appeared to remain widespread, as shown by increased recurrent polysynaptic activity, and was similar to what is seen in more immature, less refined tectal circuits. TNF-α treatment also enhanced the overall growth of tectal cell dendrites. Finally, we found that TNF-α-reared tadpoles had increased susceptibility to pentylenetetrazol-induced seizures.

**Conclusions:**

Taken together our data are consistent with a model in which TNF-α causes premature stabilization of developing synapses within the tectum, therefore preventing normal refinement and synapse elimination that occurs during development, leading to increased local connectivity and epilepsy. This experimental model also provides an integrative approach to understanding the effects of cytokines on the development of neural circuits and may provide novel insights into the etiology underlying some neurodevelopmental disorders.

## Background

Pro-inflammatory cytokines are immune signaling peptides, proteins, and glycoproteins that are secreted largely in response to immunological stimuli, such as infection or inflammation. Many of these cytokines are also found within the central nervous system, and are thought to play important signaling roles, independent of their immunological functions [1,2]. They have been implicated in a variety of neural processes, including the regulation of neuronal excitability, synaptic transmission, synaptic plasticity and, notably, they are believed to play important roles in neural development [3-6]. Interestingly, a growing body of evidence has linked abnormalities in cytokine levels, both systemic and in the central nervous system, with a variety of neurodevelopmental disorders and epilepsy [7-10]. For example, evidence of elevated levels of various pro-inflammatory cytokines, such as interleukin (IL)-6, tumor necrosis factor (TNF)-α and IL-1β have been found in the cerebrospinal fluid [11] and blood [12,13] of autistic individuals. Elevated levels of pro-inflammatory cytokine IL-8 have been detected in neonatal blood samples of individuals later diagnosed with Down syndrome [8], and increases in IL-6, IL-1β and TNF-α have also been associated with schizophrenia (see [14] for review). In a variety of animal models of epilepsy, as well as in patients suffering from epilepsy, increased elevations of pro-inflammatory cytokines IL-6, IL-1β and TNF-α have also been found in the cerebrospinal fluid, and many of these cytokines have been shown to have proconvulsant activity [9,15]. While these studies remain correlative, they suggest that elevated levels of cytokines in the central nervous system may be at least partly responsible for clinically observed developmental deficits. However, this has not been directly tested.

One common feature in many of these disorders is that they are thought to result from abnormal development of brain connectivity, rather than an overt pathophysiology or neurodegeneration [16-18]. One useful test of the hypothesis that elevated cytokine levels could contribute, at least in part, to the etiology underlying some neurodevelopmental disorders is to test whether elevated cytokine levels during development are by themselves sufficient to induce abnormalities in the formation of neural circuits. In this study we address this issue by looking at the effects of cytokines on the development of the visual system of *Xenopus laevis *tadpoles. The visual system of *Xenopus *tadpoles is frequently used as a model for the development of neuronal microcircuitry [19,20]. It is well-suited for such study, as the development of neuronal excitability, synaptic transmission, neural circuit refinement and visually guided behavior are well understood [21-25]. In this system, visual inputs originating from the retina form a topographically organized projection onto the optic tectum. This projection is initially formed via a series of molecular cues and is subsequently refined by visual experience [26,27]. Local circuits within the tectum are also known to refine during development in an activity-dependent manner [24]. Proper refinement of both retinotectal and local tectal circuits is required in order to develop normal visually guided avoidance behavior [22], and this behavior can be used to screen for abnormal development of visual circuits. In addition, a very permeable blood-brain barrier allows water-soluble pharmacological agents to be administered systemically by simply adding them to the tadpole's rearing media [28]. The *Xenopus *tadpole visual system is therefore one of the few model systems in which we can link molecular, cellular and circuit level events in a behaving animal as it goes through development.

In this study, we focused on a set of three pro-inflammatory cytokines - IL-1β, IL-6 and TNF-α - that are known to be elevated in neurodevelopmental disorders and have known effects on neuronal function. IL-1β is known to reduce current through voltage-gated Na^+ ^and Ca^2+ ^channels [29,30] as well as increase NMDA-receptor (NMDAR)-mediated currents [31], and currents through TRPV1 channels in nocioceptive neurons [32]. It also causes a decrease in spontaneous AMPA-receptor (AMPAR)-mediated transmission and interferes with normal induction of synaptic plasticity [33,34]. IL-6 is known to affect metabotropic glutamate receptor function and interfere with Ca^2+ ^release from internal stores [35]. TNF-α causes insertion of AMPARs in synaptic sites and decreases GABAA-receptor-mediated currents [36,37]. TNF-α is thought to underlie homeostatic scaling of synaptic inputs, through a β-3 integrin-mediated pathway [38,39]. It also enhances currents through tetrodotoxin-resistant Na^+ ^and voltage-gated Ca^2+ ^channels in some neurons [40,41].

Our general experimental approach consisted of rearing tadpoles in media containing elevated concentrations of the different cytokines between developmental stages 44 and 49. During this time period, several well-characterized developmental changes at the level of synaptic transmission, intrinsic excitability, retinotectal circuit refinement and visually guided behavior are known to occur [21-24]. After the rearing period, tadpoles were tested for deficits in visually guided behavior and for neuroanatomical abnormalities in the visual projection. Based on this initial assay, specific groups were selected for further electrophysiological analyses. Using this integrative approach, we aim to understand how abnormal cytokine levels may lead to neural dysfunction, which, in turn, can cascade into large-scale deficits in circuit formation and behavior.

## Results

### Behavioral screening of cytokine-reared tadpoles

**Figure 1:**
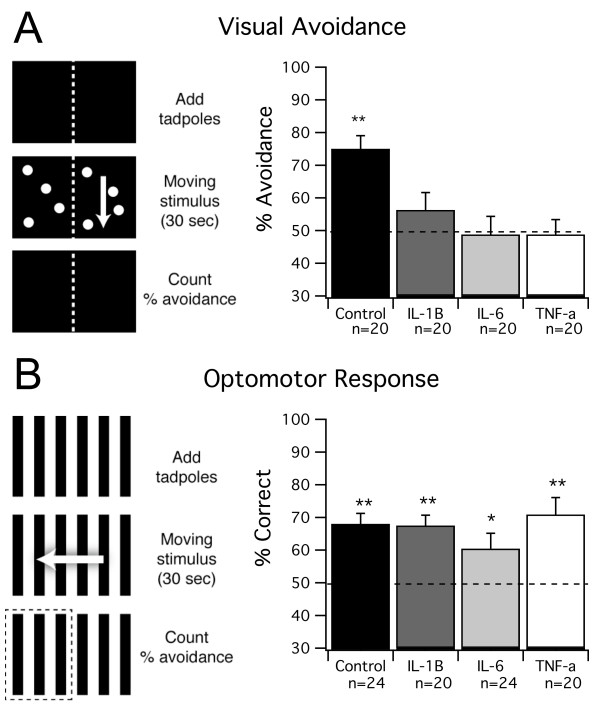
**Visual avoidance is impaired by chronic cytokine treatment**. **(A) **Control tadpoles and tadpoles reared in IL-1β, IL-6 and TNF-α were tested for normal visual avoidance behavior. This behavior requires tectal processing. Tadpoles were placed in a testing chamber with no stimulus for 30 s. The dot pattern was then presented for 30 seconds, such that the dots were drifting on one half of the tank and stationary on the other. After the stimulus, the number of tadpoles on the stationary side was counted to calculate percent avoidance (see Methods for details). All cytokine-reared groups showed impaired avoidance behavior. **(B) **Control tadpoles and tadpoles reared in IL-1β, IL-6 and TNF-α were tested for an optomotor response (OMR). This behavior does not require tectal processing. Tadpoles were placed in a testing chamber with no stimulus for 30 s. The drifting grating was presented for 30 s. After the stimulus, the number of tadpoles that swam in the direction of the grating was counted to calculate the percent correct (see Methods for details). All groups showed a significant OMR, although the OMR in the IL-6 group was slightly reduced. For *P*-values, see Results.

In order to test whether rearing tadpoles in elevated cytokine levels results in visual processing deficits, we screened cytokine-reared tadpoles using a visual avoidance test [22]. This test has been previously shown to require normal processing of visual stimuli by the tectum, and improvement in behavioral performance over development has been linked to changes in refinement of tectal circuitry; in contrast, decreased performance on this test has been directly linked to abnormal development of tectal circuits [22]. Tadpoles were reared for 10 to 12 days - roughly corresponding to the period between developmental stages 44 and 49 - in the presence of either IL-1β, IL-6 or TNF-α and compared to controls (see Methods). After treatment, tadpoles were tested using the visual avoidance test as described in the Methods. Under these rearing conditions, tadpoles raised in control media scored significantly above chance in the visual avoidance task (75 ± 4%, n = 20 trials; *P *< 0.0001; Figure 1A). In contrast, all three cytokine-treated groups demonstrated impaired avoidance behavior, performing the task at chance levels (TNF-α: n = 20 trials, 49 ± 5%, *P *= 0.789; IL-1β: n = 20 trials, 56 ± 5%, *P *= 0.1342; IL-6: n = 20 trials, 49 ± 6%, *P *= 0.82; Figure 1A). To test whether the effects of cytokine treatment were specific to tectally-mediated avoidance behavior, or due to a general impairment of all visually guided behavior or swimming ability, a control behavioral test was used. Previous work has shown that another visually guided behavior, known as the optomotor response (OMR), does not require the optic tectum, and can therefore be used as a control test for overall behavioral deficits [22,42]. In the OMR, animals swim in the direction of moving bands (see Methods). When we tested control and cytokine treated groups for the OMR, we found that IL-1β and TNF-α performed as well as controls (controls: n = 24 trials, 67 ± 3%, *P *= 0.0001; TNF-α: n = 20 trials, 71 ± 1%, *P *= 0.001; IL-1β: n = 20 trials, 68 ± 3%, *P *= 0.0001; Figure 1B). IL-6-treated tadpoles also performed significantly above chance in this task (n = 24 trials, 60 ± 5%, *P *= 0.04; Figure 1B), but at a lower level than the other groups. Taken together, these data suggest that long-term exposure to TNF-α and IL-1β results in deficits in visual behavior that are consistent with abnormal development of the retinotectal circuitry. Due to weaker OMR in the IL-6 group, we cannot rule out that the effects of IL-6 were due to non-specific effects on the health, motor ability or overall visual system function of the animal.

### Gross anatomy of the retinotectal pathway

**Figure 2:**
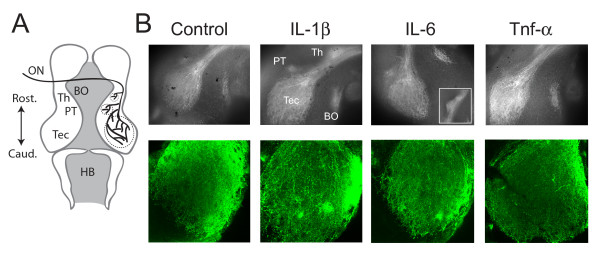
**Chronic cytokine treatment does not result in abnormalities in the gross projection pattern of central visual pathways**. (A) Diagram of the *Xenopus *tadpole brain visual projections. Visual inputs enter the tectum via the optic nerve (ON) and primarily project contralaterally to the optic tectum (Tec). Other projections are to the contralateral pre-tectal neuropil (PT), the thalamus (Th) and the basal optic neuropil (BO). Rostral and caudal directions are indicated as is the location of the hindbrain (HB). (B) Two separate examples of the visual projection are shown from Isl-2b:GFP transgenic tadpoles reared under different conditions. The top images were taken with a conventional fluorescent microscope and show the different areas. In IL-6 the thalamic projection is shown as an inset since it was outside of the field of view of the original image. The bottom images are maximum projections of confocal stacks focusing on the tectum. None of the cytokine treatments appeared to cause any gross morphological abnormalities. At least five different brains were imaged for each of the rearing conditions.

We next tested whether cytokine treatments resulted in abnormally targeted visual projections, which could account for the behavioral deficits. To do this we used a line of transgenic tadpoles expressing green fluorescent protein (GFP) driven by the Isl2b promoter, which primarily restricts expression to retinal ganglion cells. This allowed us to directly visualize the retinotectal projection using fluorescence microscopy. The pattern of innervation by the retinal ganglion cell axons enables the identification of four major targets that have been previously described [43] (Figure 2A). However, the appearance of the innervation of any one target is generally quite variable between animals, as demonstrated by the control animals; therefore, only gross defects, such as mistargeting of axons to non-visual areas, would be detected using this morphological approach. We found that cytokine treatments did not have any noticeable effect on the pattern of projections to central targets, with no evidence of projections to non-visual areas. Retinal projections to the optic tectum, the pre-tectal neuropil, the thalamus and the basal optic neuropil appeared normal (Figure 2B, top), even in the IL-6 treated group. In a subset of tadpoles, confocal images were taken of the retinotectal projection at a higher resolution to examine the pattern of innervation within the tectum. We found that in all cases tested the axons of retinal ganglion cells were targeted to the tectal neuropil, as revealed by analysis of individual confocal sections in the Z-series. Flattened images of representative Z-series are shown in Figure 2B (bottom). Thus, we conclude that any deficits in visual behavior are likely to result from either more subtle anatomical differences in the microcircuitry of the tectum, or from electrophysiological abnormalities in synaptic function in this pathway.

### Long-term effects of TNF-α on tectal cell synaptic and intrinsic properties

To further examine the effects of elevated cytokine levels during development, we chose to focus on the electrophysiological effects of TNF-α. Due to the possibility that IL-6 may have had more widespread effects on the developing tadpole, we did not examine the effects of this cytokine further. Between IL-1β and TNF-α, the effects of TNF-α on excitatory synaptic transmission are better understood; thus, we focused our attention on TNF-α, leaving IL-1β and IL-6 as the subjects of future studies. Prior work has shown that acute application of TNF-α enhances excitatory synaptic transmission in hippocampal neurons by increasing surface expression of AMPA-type glutamate receptors [36,37]. This results in enhanced frequency and amplitude of miniature excitatory post-synaptic currents (EPSCs) [36,44]. Additionally, TNF-α is known to enhance intrinsic excitability in dorsal horn neurons following nerve injury [45].

**Figure 3:**
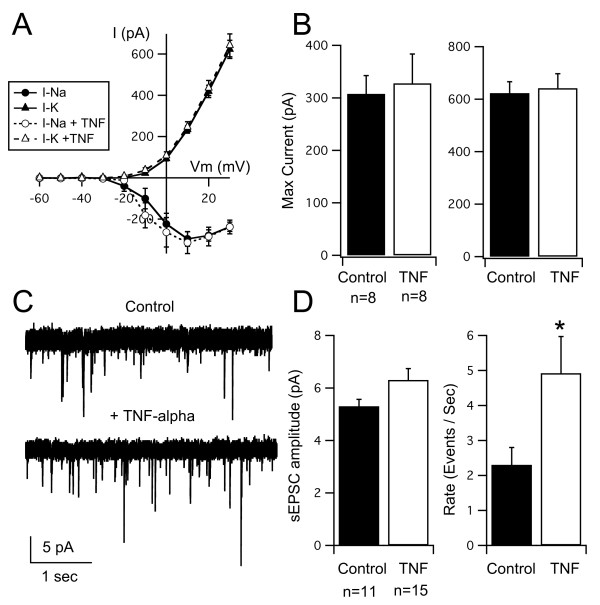
**Acute effects of TNF-α application on voltage-gated currents and spontaneous synaptic transmission in tectal neurons**. (A) I-V curves for Na^+ ^and K^+ ^currents in control tectal cells and cells acutely (approximately 1 h) treated with TNF-α. (B) Maximum peak amplitudes (pA) of Na^+ ^and K^+ ^currents from both groups show no significant differences. (C) Spontaneous EPSCs recorded from control neurons and neurons acutely (approximately 1 h) treated with TNF-α. (D) TNF-α-treated tadpoles exhibited a higher frequency of sEPSCs than controls but showed no difference in amplitude. Asterisk indicates p < 0.05, for actual *P*-values see Results.

We first tested whether acute application of TNF-α could have a direct effect on excitatory synapses in the developing tectum and whether it could alter intrinsic excitability of tectal neurons. We performed whole-cell recordings from tectal neurons in a whole-brain preparation [25]. We compared amplitudes of voltage-gated Na^+ ^and K^+ ^currents, as well as the amplitude and frequency of spontaneous AMPAR-mediated EPSCs (sEPSCs) in control brains and brains that had been exposed to TNF-α (0.5 mg/ml) for at least 1 hour. Tectal neurons exhibit voltage-gated Na^+ ^and K^+ ^currents (Figure 3A), which have been shown to directly correlate with their ability to generate action potentials [23,46]. Acute application of TNF-α did not result in changes in the current-voltage (I-V) relationship, nor the maximal amplitude of voltage-gated currents (Figure 3A, B). However, consistent with findings from hippocampal cultures, we found that acute application of TNF-α enhanced AMPAR-mediated synaptic transmission [36,37,44]. In our case, the increase was observed as an increase in sEPSC frequency and a small, but not significant, increase in amplitude (control: 2.3 ± 5 events/s, 5.3 ± 0.3 peak amplitude (pA), n = 11; TNF-α: 4.9 ± 1 events/s, 6.3 ± 0.4 pA, n = 15; frequency difference *P *= 0.038, amplitude difference *P *= 0.057; Figure 3C, D). This confirms tectal synapses can acutely respond to TNF-α, and that this pathway is active in the developing tectum.

**Figure 4:**
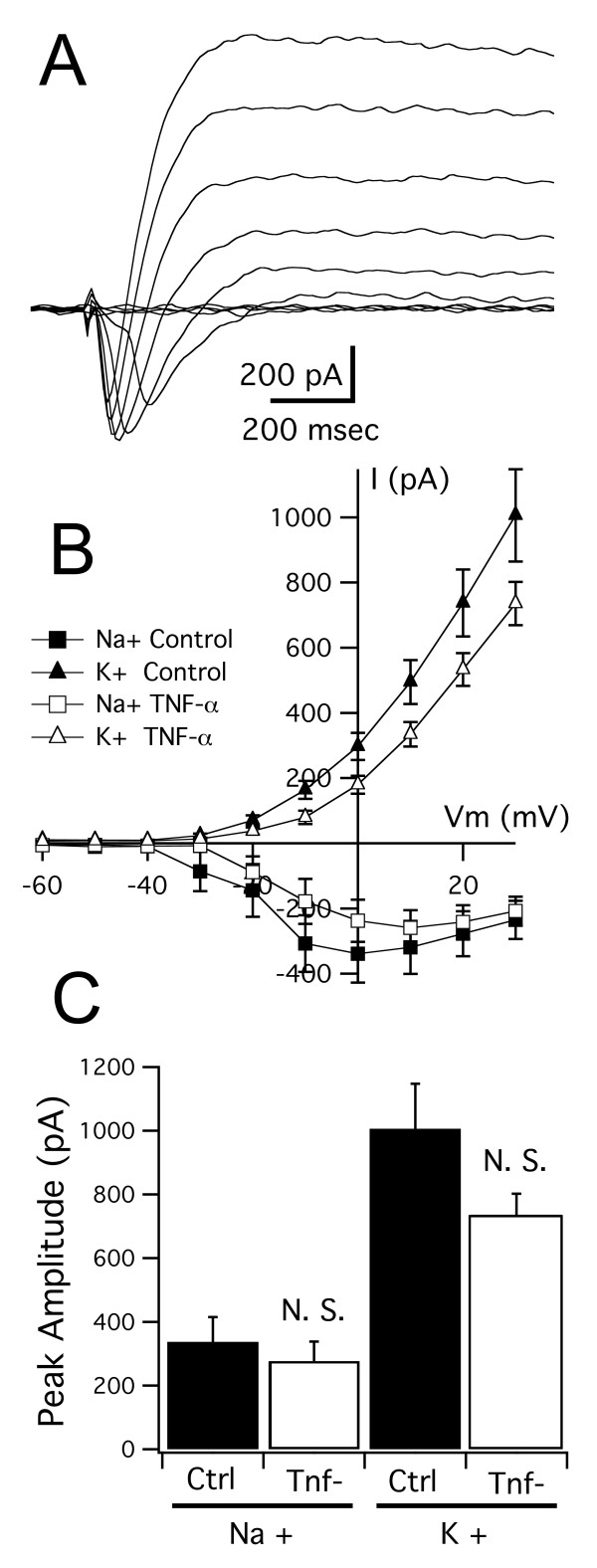
**Long-term developmental exposure to TNF-α does not alter intrinsic membrane currents of tectal neurons**. (A) Family of current traces recorded in response to a series of depolarizing steps from -60 to +30 mV. The fast inward current is a voltage-gated Na^+ ^current while the outward current is a voltage-gated K^+ ^current. (B) I-V curves for Na^+ ^and K^+ ^currents in control tectal cells and cells from TNF-α reared tadpoles. (C) Maximum peak amplitudes of Na^+ ^and K^+ ^currents from both groups show no significant differences. For *P*-values see Results. N.S., not significant.

**Figure 5:**
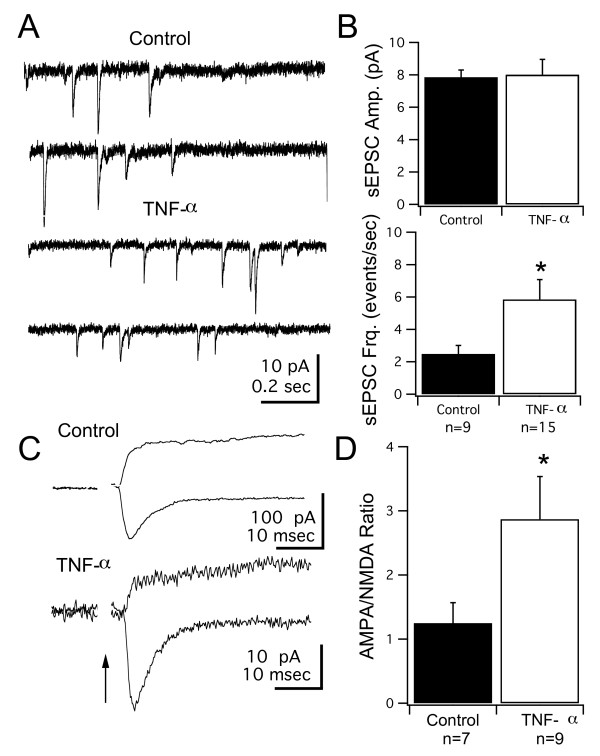
**Long-term developmental exposure to TNF-α enhances AMPAR-mediated synaptic transmission**. (A) Spontaneous EPSCs recorded from a tectal neuron in a control and a TNF-α reared tadpole. (B) TNF-α treated tadpoles exhibited a higher frequency of sEPSCs than controls but showed no difference in amplitude. (C) Sample traces showing glutamatergic EPSCs evoked by direct optic nerve stimulation and recorded at -60 and +60 mV. At -60 mV only the AMPAR-mediated response is observed, while at +60 mV both outward AMPAR- and NMDAR-mediated responses are seen. (D) The AMPA/NMDA ratio (see Methods) was significantly increased in tectal cells from tadpoles reared in TNF-α, consistent with an increase in AMPAR-mediated synaptic transmission. Asterisk indicates p < 0.05. For *P*-values see Results.

To test whether long-term elevations of TNF-α affect intrinsic excitability and synaptic properties of tectal neurons, we performed electrophysiological recordings on tectal neurons from tadpoles reared in TNF-α for 10 days, between developmental stages 44 and 49. We first looked for changes in intrinsic properties. We found no significant differences in the maximal amplitudes and current-voltage (I-V) relationships of both voltage gated Na^+ ^and K^+ ^currents between the control and TNF-α treated groups (maximum amplitude: Na^+^: 337 ± 78 pA, n = 9 versus 276 ± 62 pA, n = 9; *P *= 0.55; K^+^: 1006 ± 142 pA versus 735 ± 66 pA; *P *= 0.1;Figure 4A, B, C). We next tested the effects of TNA-α on excitatory synaptic transmission. We found that, similar to the effects of acute application, the TNF-α treated group had a significantly higher frequency of sEPSCs than controls (Figure 5A, B; TNF-α: 5.84 ± 1.24 events/s, n = 15; control: 2.48 ± 0.53 events/s, n = 9; *P *= 0.023), but no detectable difference in sEPSC amplitude (TNF-α: 8.01 ± 0.95 pA, control: 7.85 ± 0.45 pA). In our hands we have found that both the amplitude and frequency distribution of sEPSCs are not significantly different from those measured from mEPSCs recorded in tetrodotoxin (TTX) [23]. Thus, we interpret the TNF-α mediated change in sEPSC frequency as an increase in the number of functional glutamatergic synapses.

As retinotectal synapses mature, they increase the relative amount of AMPAR-mediated currents relative to NMDAR-mediated currents, as a result of 'unsilencing' of immature synapses containing only NMDAR [21,25]. This change can be detected by measuring the ratio of AMPAR-mediated currents recorded at a negative membrane potential (-60 mV) and slower NMDAR-mediated currents recorded at a positive membrane potential (+60 mV) [25,47]. This increase in the AMPA/NMDA ratio is a hallmark of synaptic maturation in excitatory synapses, both in the tectum and in other brain regions, and can be used as an index of the relative maturity of a glutamatergic synapse [21,25,47-50]. We found that TNF-α-treated tadpoles had significantly larger AMPA/NMDA ratios compared to the control group (Figure 5C, D; TNF-α: 2.87 ± 0.67, n = 9; control: 1.24 ± 0.32, n = 7; *P *= 0.0425). Because no increase in sEPSC amplitude was detected, one possibility that would account for the change in AMPA/NMDA ratio is that TNF-α is causing a premature 'unsilencing' of immature NMDAR-only synapses, rather than a strengthening of pre-existing AMPAR-containing synapses. If this possibility were true, then we would expect to see a decrease in the number of so-called silent synapses after chronic treatment with TNF-α.

**Figure 6:**
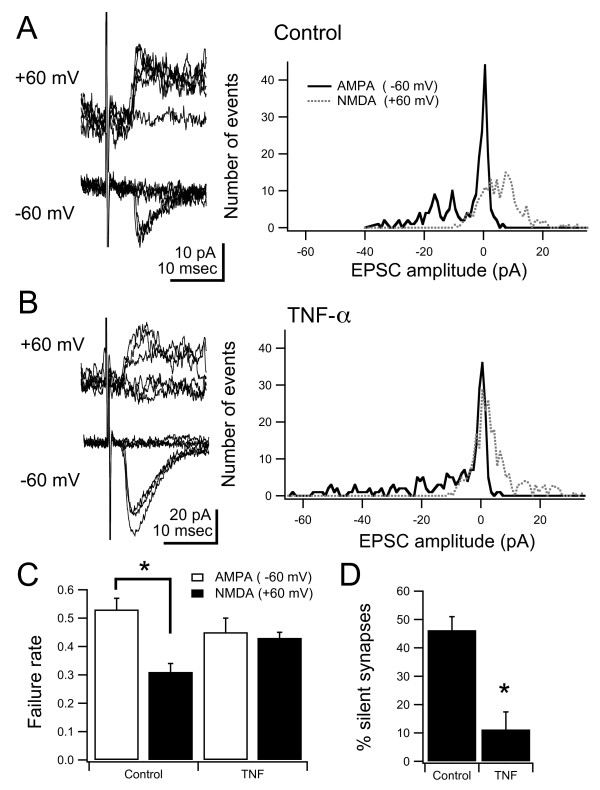
**Long-term developmental exposure to TNF-α results in a decrease of NMDAR-only synapses**. (A, B) Left: superimposed responses evoked by minimal stimulation recorded at -60 and +60 mV from control and TNF-α-treated animals. Right: histogram of all minimal stimulation AMPA and NMDA responses from control and TNF-α-treated animals. (C) Failure rates of NMDA currents were significantly lower in control animals, whereas they were not different in drug-treated animals, consistent with a decreased number of silent synapses. (D) Estimation of the percent of silent synapses in the stimulated projection was also significantly different. Asterisk indicates p < 0.05. For *P*-values see Results.

Silent synapses were measured by stimulating single retinotectal fibers, using minimal stimulation [25,47,48]. Stimuli were delivered while holding the membrane potential at -60 mV and +60 mV, and the failure rate was calculated at both potentials. At the negative membrane potential, synaptic transmission is mediated by AMPARs, while at the positive membrane potential transmission is mediated by both AMPARs and NMDARs. If a subset of the synapses in the stimulated fiber contains only NMDARs, these could only be detected at the positive membrane potential. Therefore, the synaptic failure rate would be higher at the negative membrane potential. By calculating the relative number of failures at both membrane potentials we can estimate the percent of NMDA-only synapses (see [47]). We found that after chronic treatment with TNF-α, retinotectal synapses had similar failure rates at both membrane potentials, in comparison to controls, which had relatively lower failure rates at the negative membrane potential (Figure 6A-C). Thus, retinotectal inputs treated with TNF-α contained a smaller percentage of silent synapses than controls (Figure 6D; control: 46.2 ± 4.8%, n = 9; TNF-α: 11.2 ± 6.2%, n = 9; *P *= 0.0005). One interpretation of these data is that long-term treatment of TNF-α during development results in changes consistent with enhanced maturation of excitatory synapses, including those of the retinotectal projection. Other mechanisms, such as enhanced stabilization of mature synapses or decreased synapse elimination, are also consistent with these data.

### Long-term effects of TNF-α on tectal circuits

During development, the tectal circuitry undergoes a significant amount of refinement, both in the retinotectal projection as well as in the local circuitry within the tectum. Much of this refinement involves strengthening and stabilization of some synapses, and weakening and elimination of others. In the prevailing model, synaptic maturation and AMPAR insertion is believed to stabilize existing synapses and dendritic branches [51]. Synapses that fail to mature would be retracted while mature ones would become more established, as developing circuits become increasingly organized. Based on our observations above, if TNF-α prematurely enhances the stabilization of excitatory synapses within the tectum, then we would predict that tectal circuits would remain in an unrefined state due to decreased synapse elimination. We therefore measured the effect of chronic TNF-α in the refinement of both the retinotectal projection and local tectal circuits.

**Figure 7:**
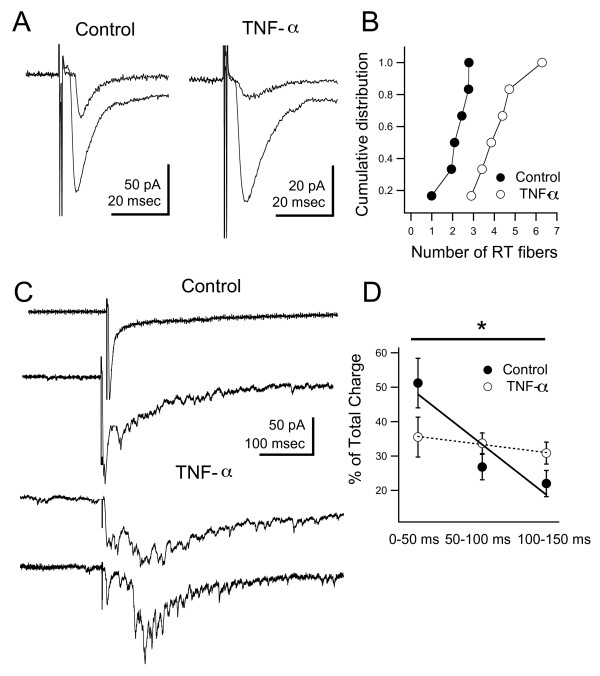
**Long-term developmental exposure to TNF-α results in altered tectal circuitry**. (A) Sample retinotectal synaptic currents in response to minimal and maximal electrical stimulation of the optic nerve. (B) The number of retinotectal (RT) inputs innervating a single tectal cell was estimated by dividing the maximal response by the minimal response. TNF-α-treated tadpoles show an increased number of retinotectal inputs per cell, consistent with lack of developmental refinement. (C) Maximal stimulation of the optic chiasm results in a prolonged barrage of polysynaptic activity driven by local intratectal circuits. The first peak of the response is the monosynaptic retinotectal input. Two sample traces are shown for both control and TNF-α reared tectal neurons. Notice that the duration and distribution of the recurrent activity is longer and slower in the TNF-α reared tadpoles. (D) Quantification of the time course of the polysynaptic activity. Synaptic charge was measured over the first three 50-ms bins following the onset of the response. Data were plotted as percent of the total synaptic charge during the entire time period. Grouped data were then fitted to a line using linear regression. Neurons from TNF-α reared tadpoles show significantly slower decay of the polysynaptic response, indicating increased intratectal interconnectivity. Asterisk indicates p < 0.05. For *P*-values see Results.

To assess the effects of TNF-α on refinement of the retinotectal projection, we estimated the number of retinotectal fibers innervating a single tectal cell. Over development, the number of retinotectal fibers innervating a single tectal cell is known to decrease. This calculation was done by first measuring the amplitude of the synaptic response evoked by single-fiber stimulation. The stimulus intensity was then increased until the retinotectal synaptic response leveled-off at a maximal value. We then divided the maximal amplitude by the minimal amplitude to get an estimate of the number of retinotectal fibers innervating a single tectal cell (Figure 7A). Typically, one can use the number of discrete jumps in evoked response amplitude, as the stimulus intensity is increased, to calculate the number of inputs to a given cell. However, due to the accumulated amplitude variability of single fiber responses, it was not possible to always detect discrete jumps. Therefore, we followed the above method to estimate fiber number. It should be noted that one caveat with this approach is that it assumes that all inputs have similar amplitudes, and therefore it may over- or underestimate the number of fibers. Nevertheless, the relative changes in fiber number over development should still be detectable with our method. We found that tectal neurons in tadpoles treated with TNF-α received significantly more retinotectal inputs than controls (Figure 7B; TNF-α: 4.26 ± 0.49, n = 6; control: 2.17 ± 0.27, n = 6; *P *= 0.007). These data are consistent with a lack of refinement of the retinotectal projection.

In the *Xenopus *tectum, a large proportion of excitatory synapses originate from local tectal circuits. Activation of these local circuits by afferent activity results in persistent recurrent activity within the tectum [24]. This recurrent activity becomes increasingly organized during development. Between stages 44 and 49, the temporal pattern of recurrent activity becomes more temporally coherent, occurring at shorter latencies following the afferent input, and shows less trial-to-trial variability. This process is dependent on neural activity and is consistent with a spike-timing dependent plasticity rule where certain local inputs are weakened while others are strengthened [24]. We predict that if TNF-α treatment results in premature maturation of glutamatergic synapses in the tectum, then this process by which local circuitry becomes organized would be disrupted, resulting in a lack of refinement of local circuitry and an increase in local interconnectivity among tectal neurons. To test this we compared recurrent activation of tectal neurons in control and TNF-α reared tadpoles. Recurrent activity was evoked by maximally stimulating the optic nerve in the presence of inhibitory blocker picrotoxin (PTX) [24]. In control stage 49 tadpoles, recurrent activity was detected at a short latency following the direct optic nerve response, often blending in with the monosynaptic retinotectal input, consistent with prior studies (Figure 7C, top traces). In contrast, in the TNF-α treated group, the recurrent activity was much more pronounced and occurred at longer latencies, similar to what is observed in immature tadpoles (Figure 7C, bottom traces). The temporal profile of the recurrent activity was quantified by calculating the total synaptic charge in three adjacent 50-ms time bins and calculating the decay rate by linear regression through all three time points [24]. We found that the synaptic charge rapidly decayed after the first 50 ms in the control group, whereas in the TNF-α-treated group the synaptic charge remained fairly constant over the first 150 ms and beyond (Figure 7D). This difference was statistically significant (*P *= 0.05, F-test). One alternative possibility is that the prolonged recurrent synaptic activity could be due to enhanced intrinsic excitability of tectal neurons. However, as shown in Figure 3, chronic treatment with TNF-α does not result in increased intrinsic excitability of tectal neurons. Thus, when taken together these data suggest that long-term elevations in TNF-α levels prevent developmental refinement of retinotectal synapses and local tectal circuits, possibly due to enhanced stabilization of excitatory synapses.

**Figure 8:**
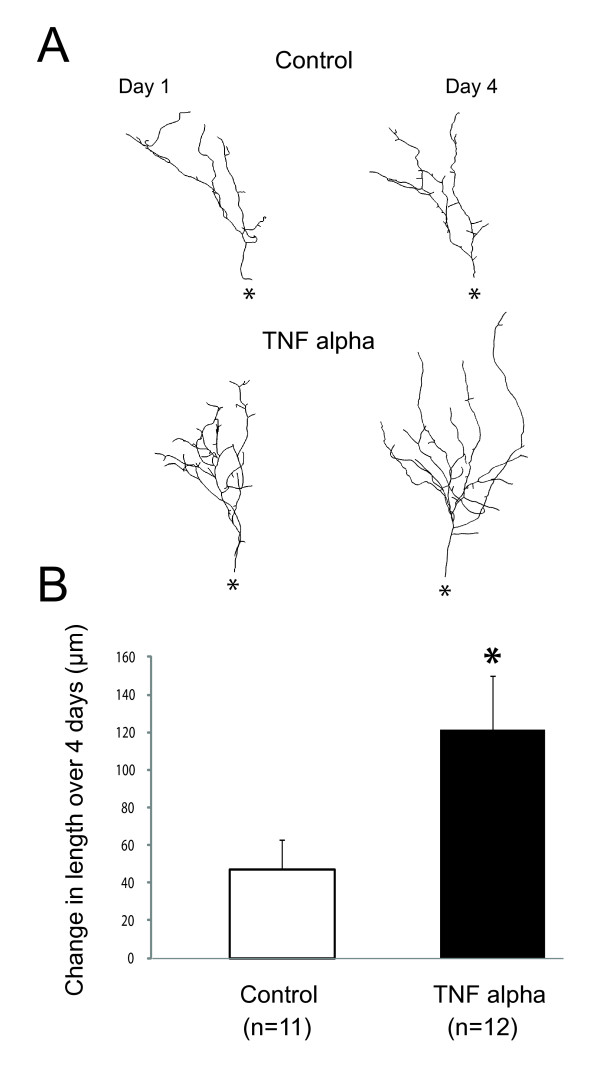
**Long-term developmental exposure to TNF-α results in enhanced growth of tectal cell dendrites**. (A) Representative images of tectal cell dendrites imaged before drug treatment and after chronic treatment with TNF-α. The TNF-α cells are compared with sham-treated controls. Asterisk indicates location of cell body. (B) TNF-α results in significantly enhanced growth rate of tectal cell dendrites. Asterisk indicates p < 0.05. For *P*-values see Results.

One further test of this hypothesis would be that if TNF-α treatment results in premature stabilization of synapses onto tectal neurons, then tectal neuron dendrites should reflect this effect. Tectal dendrites grow through a balance of branch addition and retraction [51]. New dendritic branches are added continuously, and if functional synapses are established, then these branches are stabilized. If no functional synapses are made, then branches eventually retract. Ultimately, as synapses mature and become stabilized, the dendritic arbor grows in size as more added branches are stabilized than retracted. We predict that we should see a greater amount of dendritic growth in TNF-α-treated tadpoles due to enhanced branch stabilization as well as overall larger dendritic arbors. To test this, individual tectal neurons were transfected via electroporation with GFP (see Methods) and chronically treated with TNF-α. Cells were imaged on the first and last day of treatment and the amount of dendritic growth was calculated. We found that tectal neurons in TNF-α-treated tadpoles had increased dendritic growth rates and larger dendritic arbors than controls (Figure 8; control: length, 605 ± 49 μm, and growth, 47.3 ± 14.5 μm/4 days, n = 11; TNF-α: length, 834 ± 40 μm, and growth, 121.2 ± 28.8 μm/4 days, n = 12; *P *= 0.006 for length, *P *= 0.039 for growth). This observation is consistent with enhanced synapse stabilization.

### Long-term elevations of TNF-α enhance seizure susceptibility

**Figure 9:**
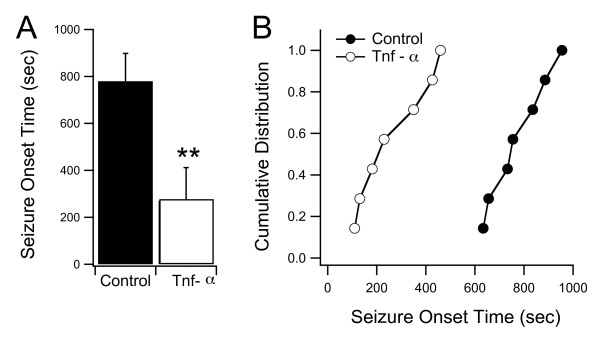
**Long-term developmental exposure to TNF-α results in increased seizure susceptibility**. (A) Tadpoles were exposed to 10 mM of the convulsant agent PTZ in their rearing media and the latency to seizure onset was calculated (see Methods). TNF-α reared tadpoles consistently showed a faster seizure onset time than their control clutchmates. (B) Cumulative probability distribution of seizure onset times for individual tadpoles in both groups. Asterisk indicates p < 0.05. For *P*-values see Results.

One possible consequence resulting from increased recurrent circuitry within the tectum is that this circuitry may facilitate the generation of seizures. Interestingly, some neurodevelopmental disorders are often associated with an increased tendency toward epilepsy [18,52-55]. Thus, we tested whether long-term treatment with TNF-α also resulted in increased seizure susceptibility. Tadpoles and embryonic zebrafish have been successfully used in the past as epilepsy models, particularly because of the ease of administering convulsant agents through the rearing media and easily quantifiable seizure behavior [56,57]. To test for seizure susceptibility, we exposed control and TNF-α treated tadpoles to 10 mM pentylenetetrazol (PTZ), a commonly used convulsant agent. We then calculated the time interval between initial PTZ exposure and the time when tadpoles exhibited clearly defined seizure behavior (see Methods). We found that the TNF-α-treated group seized significantly sooner than the control group. The average seizure onset time for the TNF-α-treated tadpoles was 270 ± 142 s (n = 7), while in the control group it was 779 ± 119 s (n = 7) (*P *< 0.0001; Figure 9; Additional file 1).

## Discussion

Our data indicate that elevated levels of pro-inflammatory cytokines IL-1β, IL-6 and TNF-α during early development of the *Xenopus *visual system result in impairment of visually guided avoidance behavior. This behavior is known to require normal maturation of retinotectal circuits. Both IL-1β and TNF-α appear to selectively disrupt tectally-mediated behavior but not the OMR, a visual behavior largely independent of tectal function. All three cytokines tested did not appear to have noticeable effects on the gross anatomical projection pattern of visual inputs. TNF-α was tested for long-term effects on synaptic transmission in the tectum, and it was found to result in increased frequency of sEPSC, enhanced AMPA/NMDA ratios and decreased number of immature, NMDA-only synapses when compared to developmentally matched controls. The TNF-α treated group also lacked normal refinement of the retinotectal projection as well as refinement of intratectal circuitry, as assayed electrophysiologically. This lack of refinement resulted in abnormal patterns of recurrent synaptic activity. Anatomically, individual tectal neurons were observed to have enhanced dendritic growth rates after treatment with TNF-α Finally, the TNF-α treated group had an increased susceptibility to PTZ-induced seizures. Taken together, our data are consistent with a working model in which TNF-α results in premature maturation of excitatory synapses within the tectum. This enhances the stabilization of tectal synapses, and impairs normal synapse elimination that occurs during refinement of the retinotectal projection and local tectal circuits. As a result, tectal cells will have increased local interconnectivity, which would cause deficits in visual processing as well as an increased tendency to seizures.

### Abnormal maturation of retinotectal circuits

Our data indicate that elevated cytokine levels during development are sufficient to induce abnormalities in tectal microcircuitry as shown by behavioral studies. Furthermore, our electrophysiological data suggest that the direct effect of TNF-α on excitatory synaptic transmission may be responsible for preventing the normal maturation of tectal microcircuitry. Accumulating evidence from the developing tectum and other brain regions has suggested that functional and anatomical maturation of neural circuits are concurrent, and that functional synaptic maturation is a critical step in stabilizing developing synapses. This idea is known as the 'synaptotropic hypothesis' [51,58]. In our experiments, the increase in AMPAR-mediated synaptic transmission appears to result in premature maturation of tectal synapses during a time in development when retinotectal inputs and local circuits within the tectum undergo significant activity-dependent refinement [22-24]. The premature maturation of tectal synapses might stabilize existing circuits before they get a chance to refine, resulting in their abnormal development. This is supported by the fact that visually guided avoidance behavior, which is known to require normal maturation of both retinotectal and intratectal circuits, is impaired in tadpoles reared with elevated TNF-α levels. Furthermore, the combined electrophysiological findings that tectal neurons in TNF-α reared tadpoles have a greater number of retinotectal inputs, that their recurrent activity is similar to that seen in immature tadpoles and that the frequency of sEPSCs is increased (suggesting an increase in the number of tectal synapses) provide additional support for this model. Finally, the anatomical observation that the rate of dendritic growth in tectal cells of TNF-α-treated tadpoles is greater further strengthens this interpretation, and is consistent with existing models of dendritic development [59,60].

Could the other cytokines tested be acting in a similar manner? Because IL-6 may have also had an effect on the OMR, we cannot rule out that its behavioral deficit is due to a more general visual or developmental deficit. However, IL-1β did have a specific effect on visual avoidance. IL-1β is known to affect long-term potentiation, reduce AMPAR-mediated transmission and alter NMDAR function [31,33,34]. These effects could also result in abnormal development of tectal circuitry, ultimately affecting behavior. Future studies will focus on the electrophysiological effects of IL-1β and IL-6.

### Increased seizure susceptibility

Another interesting finding in our study is that TNF-α reared tadpoles have a significantly higher seizure susceptibility than controls (Figure 9). It is known that cells in the optic tecta of *Xenopus *tadpoles and zebrafish embryos show distinct seizure-like activity when presented with a convulsant agent [56,57], and it has been proposed that activity in the tectum may, at least in part, be involved in promoting seizures. Our finding is consistent with an interpretation in which increased seizure susceptibility occurs in response to increased recurrent activity within the tectum, and perhaps in other circuits throughout the tadpole central nervous system. Our data are in alignment with one common model for epileptogenesis in which increased seizure susceptibility is due to the increased formation of recurrent excitatory circuits [18]. Alternatively, the increased seizure susceptibility may also be due to a decrease in the amount of inhibition relative to excitation [61]. Prior studies have shown that TNF-α can also decrease the amount of GABAergic transmission in the hippocampus, which would also help promote seizure activity [37]. It remains to be determined whether this complementary mechanism is also present in our system. A final alternative is that increased seizure susceptibility may be due to enhanced intrinsic excitability of tectal cells; however, our data do not support this interpretation.

### A model for neurodevelopmental disease

Several neurodevelopmental disorders, including autism, Down syndrome and schizophrenia have been associated with abnormal maturation of neuronal microcircuitry [16,17] and they have also been associated with elevated levels of pro-inflammatory cytokines [7,8,14]. Interestingly, one common feature of several neurodevelopmental disorders is also the development of epilepsy [18,52-54]. This finding has also been borne out in animal disease models (for example, [55]). How do our findings relate to these observations?

We find that cytokine-reared tadpoles exhibit some of the features associated with a number of neurodevelopmental diseases and disease models: abnormal development of neuronal microcircuits, lack of developmental pruning, and increased tendency to seizures. However, many of these disorders have been primarily associated with a wide variety of genetic causes, and animal models interfering with these different genes appear to replicate many of the phenotypic aspects of the various disorders [55,62-65], although it has also been suggested that these multiple disorders may also share common biological pathways [66]. One common theme that emerges from these findings is that any genetic alterations that interfere either positively or negatively with the normal formation and function of synapses is likely to result in neurodevelopmental disorders associated with malformation of neural microcircuitry. Our data are consistent with this picture, and present a possible mechanism by which cytokines, functioning at the synaptic level, might exacerbate any functional abnormalities caused by underlying genetic defects. While we show that elevated TNF-α alone is sufficient to induce some neurodevelopmental abnormalities, we believe that it is unlikely that elevated cytokines by themselves will be the sole cause of neurodevelopmental disorders. Rather, TNF-α and other cytokines might possibly work in tandem with other underlying genetic mechanisms, such as the ones described above, and perhaps lead to varying degrees of severity observed in these disorders. Alternatively, the underlying genetic mechanisms may make the nervous system more susceptible to the possible neurodevelopmental effects of chronic cytokine elevations or vice versa.

## Conclusions

Taken together our studies present a novel integrative model to study neuro-immune interactions. Our assays test multiple parameters of development ranging from behavior, neuroanatomy and whole-brain electrophysiology. We show that a select group of pro-inflammatory cytokines can lead to developmental abnormalities consistent with inappropriate formation of neuronal microcircuits. While we tested only three cytokines, our assays could be scaled up to study a much broader range of immune molecules and combinations of molecules, providing for a powerful large-scale assay for abnormal neural development. We believe our findings might provide valuable insight into novel therapeutic approaches for the treatment and understanding of neurodevelopmental disorders.

## Methods

All of our experiments were carried out in accordance with Institutional Animal Care and Use Committee standards. The tadpoles were staged according to Nieuwkoop and Faber developmental stages (as described in [23]) and generally reached stage 42 5 to 6 days post-fertilization (dpf), stages 44 to 46 at 7 to 12 dpf, and stage 49 at 18 to 20 dpf under our rearing conditions. Both wild-type and experimental *Xenopus laevis *tadpoles were raised on a 12 hour light/dark cycle at 20 to 22°C.

### Cytokine treatment

Tadpoles were reared in 15-ml wells of a 6-well plate containing 5 to 6 ml of IL-6 (0.065 μg/ml), IL-1β (0.10 μg/ml), or TNF-α (0.50 μg/ml). Treatment began at stage 44 (approximately 7 dpf) and media was exchanged every 2 to 3 days. Tadpoles were treated until they reached stages 48 to 49, about 18 to 20 dpf. A maximum of four tadpoles were reared in each well, and control tadpoles, which were derived from the same clutch as the treated groups, were also reared in the same wells and rearing media volume. TNF-α was purchased from Sigma (St. Louis, MO, USA). IL-1β and IL-6 were purchased from R&D Systems (Minneapolis, MN, USA).

### Avoidance behavior and optomotor response testing

Behavioral testing was performed as described in Dong *et al*. [22]. In brief, tadpoles were tested in a clear-bottomed, rectangular tank (16 × 10 cm) filled to an approximate depth of 1.5 cm with rearing media at room temperature (20 to 22°C). The tank was rested atop the screen of a CRT monitor. The sides and top of the tank were darkened. The behavioral test sequence for the avoidance response was as follows (Figure 1A). A group of tadpoles was placed in the center of the tank over a black screen, and the tank was covered for 30 seconds. After this period, white dots with a 2-mm radius were presented for 30 seconds such that the stimulus pattern in one-half of the tank was drifting while the other remained stationary. The direction of drift was orthogonal to the length of the tank. At the end of the stimulus period, the number of tadpoles on the stationary side was noted, and the percent of tadpoles on the stationary side was calculated for each trial. Control tadpoles will tend to aggregate in the stationary side. To test for the OMR, a similar procedure was followed except that alternating black and white bars drifted along the length of the tank, and the number of tadpoles on the side toward which the bars drifted was counted (Figure 1B). The stimulus patterns were generated using a custom-written MATLAB (MathWorks Ltd, Natik, MA, USA) script using the Psychophysics Toolbox extensions [67].

Tadpoles were tested in groups of four, and each group of four tadpoles was counted as one trial, and the percentage of tadpoles on the stationary versus moving side were calculated for each trial. The percent avoidance was calculated as the average percent of tadpoles in the stationary side after multiple trials. A one-sample *t*-test was used to test whether the percentage of tadpoles on the stationary side was different from a random distribution in which tadpoles are evenly split between the stationary and non-stationary sides (see [22]). This design allows us to test for significance for individual groups under individual experimental conditions and better reflects the trial-to-trial variability. All error values are standard error of the mean.

### Neuroanatomy of the retinotectal projection in Isl2b:GFP tadpoles

Isl2b:GFP tadpoles are transgenic tadpoles expressing GFP driven by the Isl2b promoter (also known as Isl3, gift of Chi-Bin Chien, University of Utah), which primarily drives expression in retinal ganglion cells [68]. Isl2b:GFP tadpoles were obtained via natural mating between an Isl2b:GFP+ male and a wild type female *X. laevis*. After fertilization, the embryos were placed in dishes of Steinberg's solution and kept at 19 to 20°C. The tadpoles were screened for GFP expression and fed on day 5 post-fertilization. On day 6, at stage 44 the tadpoles were treated with cytokines as described earlier. On the completion of the tenth day of cytokine treatment, the tadpoles were euthanized and fixed in paraformaldehyde. The brains were then dissected from the tadpoles and placed in phosphate-buffered saline in preparation for whole mount immunostaining. The tissue was permeabilized using 1% Triton X-100 and 0.1% bovine serum albumin in phosphate-buffered saline (PBT), and blocking was done with 10% normal goat serum in PBT. The primary antibody incubations were done at 4°C O/N using 1:1,000 rabbit αGFP (Torrey Pines, Del Mar, Ca, USA) to label retinal ganglion cell axons. The secondary antibody incubation was done at room temperature for 4 hours using 1:200 α rabbit Alexa-488. The brains were then mounted on coverslips and pictures were taken on the fluorescent microscope. For high resolution images an Olympus confocal microscope was used.

### Electrophysiology

For whole-brain recordings, tadpole brains were prepared as described by Wu *et al*. [25] and Aizenman *et al*. [46]. Tadpoles were first anesthetized in 0.01% tricaine methane sulfonate (MS-222) and pinned to facilitate brain isolation. The isolation was performed in HEPES-buffered extracellular saline (in mM: 115 NaCl, 2 KCl, 3 CaCl_2_, 3 MgCl_2_, 5 HEPES, 10 glucose, and 0.1 picrotoxin, pH 7.2; osmolarity, 255 mOsm). The brains were opened along the dorsal midline and pinned to a submerged block of Sylgard in a recording chamber maintained at room temperature (approximately 20 to 22°C) with continuous perfusion of external saline. Using a broken glass recording pipette, the ventricular membrane surrounding the tectum was scraped, exposing tectal neuron somata for recording. A bipolar stimulating electrode (FHC, Bowdoin, ME, USA) was placed on the optic chiasm to stimulate retinal ganglion cell axons. Visualized whole-cell voltage-clamp recordings were made using glass micropipettes (8 to 12 MΩ) filled with K^+ ^gluconate intracellular saline (in mM: 100 K-gluconate, 8 KCl, 5 NaCl, 1.5 MgCl_2_, 20 HEPES, 10 EGTA, 2 ATP, and 0.3 GTP, pH 7.2; osmolarity, 255 mOsm).

Because developmental variability exists along the rostrocaudal axis [25], we consistently recorded from cells located in the middle third of the tectum. Electrophysiological responses were measured with an Axopatch 200B amplifier (Axon Instruments, Sunnyvale, CA, USA), digitized at 10 kHz using a Digidata 1322A analog-to-digital board, and acquired using pClamp 8 software (Axon Instruments, Sunnyvale, CA, USA). Leak subtraction was done in real time using the acquisition software. Data were analyzed using AxographX software (AxoGraph Scientific, Sydney, Australia).

I-V plots were generated by measuring either the peak inward or the peak outward current in response to a series of depolarizing pulses, ranging from -60 to +20 mV [23]. Spontaneous synaptic events were collected and quantified using a variable amplitude template [69]. Evoked synaptic responses were obtained by directly stimulating the optic chiasm with 200-μs-long current pulses.

To calculate AMPA/NMDA ratios, we collected evoked synaptic responses at -60 mV to measure AMPAR-mediated EPSCs and at +60 mV to measure NMDAR-mediated EPSCs. The peak AMPA response was used to calculate the AMPAR component and the amplitude between 15 and 17.5 ms post response onset was used to calculate the slower NMDA component. Although this time window may not be an accurate absolute measure of NMDA peak response, the method still allows us to calculate relative changes in AMPA/NMDA ratio. This method was successfully used to detect developmental changes in the AMPA/NMDA ratio in this and other preparations [25,49,50]. To calculate the percentage of silent synapses, we used minimal stimulation at -60 mV and +55 mV. Failure rates at both holding potentials were measured and used to calculate the percentage of silent synapses present as described in [25,47]. All statistics used a *t*-test, unless otherwise specified, and were done using InStat or Prism software (GraphPad, La Jolla, CA, USA). Error bars are standard error of the mean.

### Single-cell electroporation, imaging and reconstruction

Micropipettes were filled with a solution containing a plasmid encoding farnesylated enhancedGFP (EGFP-F) at a concentration of 5 μg/μl. Tadpoles (developmental stage 43 to 45) were anesthetized by immersion in 0.02% MS-222. The micropipette was positioned in the superficial tectum under microscope guidance using a Narashige micromanipulator, a 0.5-s 200 Hz burst of 1-ms square wave pulses was then applied using a grass SD9 stimulator as described in Ruthazer et al., 2005 E.S. Ruthazer, K. Haas, A. Javaherian, K.R. Jensen, W.C. Sin and H. Cline, Vivo Time-lapse Imaging of Neuronal Development. In: R. Yuste and A. Konnerth, Editors, Imaging in Neuroscience and Development: A Laboratory Manual, Cold Spring Harbor Laboratory Press, Cold Spring Harbor, N.Y. (2005), pp. 191-204 [70]. Animals were then returned to their rearing tanks for 1 to 2 days to allow expression of the constructs before imaging experiments.

For imaging, tadpoles were anesthetized by immersion in 0.02% MS-222 and immobilized in Sylgard chambers (Dow Corning, Corning, NY, USA) carved to fit their body shapes with a cover glass on top. Single cells were then visualized and imaged on a custom-built two-photon microscope in 1-μm z-steps. After the first image animals were placed into rearing medium containing 0.5 μg/ml recombinant human TNF-α (BioShop Canada Inc., Burlington, ON, Canada). This solution was then replaced 2 days later. On the fourth day cells were imaged again. Three-dimensional tracings of imaged neurons were made with Imaris software (Bitplane, Zurich, Swizerland).

### Seizures

Seizures were induced in the tadpoles by the bath application of 10 mM PTZ, a known convulsant, for 20 minutes [56]. Animals initially undergo increased motor activity and turning behavior, followed by characteristic C-shaped tail bends, which become more frequent as PTZ exposure time increases [57]. Seizure onset time is measured as the time when the animals first show clear C-shaped tail bends. This is typically followed by a brief periods of immobility. All seizures were imaged with a Series 2 iSight external camera (Apple Computer, Cupertino, CA, USA) mounted vertically overhead. Seizure onset times were determined by offline video analysis using QuickTime software (Apple Computer) and analysis was done blind to treatment. Statistics were calculated with a Mann-Whitney test.

## Abbreviations

AMPAR: AMPA receptor; dpf: days post-fertilization; EPSC: excitatory post-synaptic current; GFP: green fluorescent protein; IL: interleukin; NMDAR: NMDA receptor; OMR: optomotor response; pA: pico amperes; sEPSC: spontaneous EPSC; TNF: tumor necrosis factor.

## Competing interests

The authors declare that they have no competing interests.

## Authors' contributions

RHL performed the behavioral screen and the majority of the electrophysiology. He also performed data analysis and participated in producing the final manuscript. EAM performed the neuroanatomy experiments and interpreted the results. NS performed and analyzed the functional imaging experiments. MRB performed and analyzed the seizure experiments. KED performed additional electrophysiological experiments. ESR oversaw the functional anatomy. NM-A oversaw the gross neuroanatomical experiments and provided the Isl2b:GFP tadpoles. CDA conceived of the study, participated in its design and coordination, performed some electrophysiology experiments and helped draft the manuscript and prepare the figures.

## Supplementary Material

Additional file 1 **Progression of PTZ-induced seizures in control and TNF-α reared tadpoles**. Movie shows a control well and a well containing TNF-α reared tadpoles. Tadpoles are stage 48 and are clutchmates. PTZ is introduced at t = 0. At t = 20 s normal swimming behavior is observed in both groups. At t = 5:30 minutes the TNF-α group is showing periods of increased locomotor activity. At t = 7:30 minutes the TNF-α group is undergoing clear seizure behavior characterized by extreme tail bends and fast turning behavior followed by periods of immobility. At t = 10 minutes the TNF-α group continues to seize while the control group begins to exhibit some increased locomotor activity. At t = 18 minutes both groups exhibit regular seizures interspersed with periods of immobility. Click here for file
